# Reduced Angiopoietin Factor 2 Levels Are Correlated with Better Cardiac Function and Prognosis in Valvular Heart Disease

**DOI:** 10.21470/1678-9741-2021-0364

**Published:** 2023

**Authors:** Jian Hou, Xiaolin Huang, Liqun Shang, Guangxian Chen, Huawei Wu, Zhongkai Wu, Suiqing Huang

**Affiliations:** 1 Department of Cardiac Surgery, The First Affiliated Hospital of Sun Yat-sen University, Guangzhou, Guangdong Province, People’s Republic of China.; 2 NHC Key Laboratory of Assisted Circulation, Sun Yat-sen University, Guangzhou, Guangdong Province, People’s Republic of China.; 3 Department of Neurobiology, Physiology and Behavior, College of Biological Sciences, University of California, Davis, California, United States of America.

**Keywords:** Valvular Heart Disease, Angiopoietin 1, Angiopoietin 2, Heart Valve Diseases, Inflammation, Cytokines

## Abstract

**Introduction:**

There are few circulating biomarkers for valvular heart disease. Angiopoietin (Ang) 1, Ang2, and vascular endothelial growth factor are important inflammation-associated cytokines. The aim of this study was to investigate the clinical significance and association of Ang1, Ang2, and vascular endothelial growth factor in valvular heart disease.

**Methods:**

This is a retrospective study; a total of 62 individuals (valvular heart disease patients [n=42] and healthy controls [n=20]) were included. Plasma levels of Ang1, Ang2, and vascular endothelial growth factor were detected by enzyme-linked immunosorbent assays. We retrospectively collected the baseline characteristics and short-term outcomes; logistic regression was performed to identify predictor for short-term mortality.

**Results:**

Ang2 was significantly decreased in the valvular heart disease group compared with the healthy control group (P=0.023), while no significant difference was observed in the Ang1 and vascular endothelial growth factor levels. The Ang2 level of New York Heart Association (NYHA) I/II patients — but not NYHA III/IV patients — was significantly decreased compared with that of healthy control individuals (NYHA I/II: P=0.017; NYHA III/IV: P=0.485). Univariable logistic regression analysis indicated that Ang2 was a significant independent predictor for short-term mortality (odds ratio 18.75, P=0.033, 95% confidence interval 8.08-102.33). Ang1 was negatively correlated with Ang2 (P=0.032, Pearson’s correlation coefficient =-0.317) and was positively correlated with vascular endothelial growth factor (P=0.019, Pearson’s correlation coefficient = 0.359).

**Conclusion:**

Ang2 might serve as a therapeutic and prognostic target for valvular heart disease.

## INTRODUCTION

Valvular heart disease (VHD) is an important cause of morbidity and mortality, with a prevalence that is predicted to increase dramatically because of the increase in life expectancy in middle-income and high-income nations. Despite the wider appreciation of the emerging importance of VHD, the mechanisms underlying its development are poorly understood, and the role of circulating biomarkers in guiding clinical management in individual patients is relatively unexplored.

Angiopoietin (Ang) 1 and 2, secreted 70-kDa glycoproteins and well-known members of the Ang-Tie pathway, have been demonstrated to participate in a number of cardiovascular diseases^[1-3]^. Ang2 mediates vascular leakage and inflammation, while Ang1 has antipermeability, anti-inflammatory, and cardioprotective effects^[4-7]^. Higher plasma Ang2 levels were predictive of myocardial infarction^[8,9]^ and stroke recurrence^[10]^ and were independent of traditional risk factors. In acute myocardial infarction, the extent of myocardial damage correlated with serum Ang2 and Ang2/Ang1^[11]^, indicating that Ang1 and Ang2 are potential biomarkers of the severity of cardiac disease.

Vascular endothelial growth factor (VEGF) has been shown to act as a proinflammatory cytokine by inducing the expression of adhesion molecules that cause leukocytes to bind endothelial cells^[12,13]^. Moreover, increased VEGF levels were associated with an imbalance of Ang1 and Ang2^[14]^, suggesting the complicated connection of these inflammation-associated cytokines. However, investigations on the correlation of VEGF and Ang have mostly focused on coagulation, and few have been related to inflammation.

Given that VHD is characterized by an ongoing inflammatory cellular response, we hypothesized that Ang1, Ang2, and VEGF play important roles in the progression and prognosis of VHD. To explore this hypothesis, plasma levels of Ang1, Ang2, and VEGF in VHD patients and healthy individuals were measured.

## METHODS

### Participants’ Clinical Information

Forty-two subjects with VHD were recruited between March 2018 and March 2019. All patients underwent a valve replacement procedure and recovered well. Twenty subjects without VHD and asymptomatic cardiovascular events were recruited as a healthy control group.

The exclusion criteria included age < 18 years old, recent-onset acute cardiovascular or cerebrovascular events, trauma, or surgery, coronary artery heart disease, pregnancy, acute or chronic infection, autoimmune disease, malignant tumor, and pulmonary, hepatic, or renal dysfunction. Clinical data of the patients were reviewed and collected. The study was approved by the clinical research and experimental animal ethics committee ([2017]157), and all patients enrolled in this study provided informed consent prior to participating in the study.

### Blood Sample Collection and Detection

Blood samples from VHD patients and healthy controls were obtained from forearm veins using 6.0-mL vacutainer tubes (BD, Plymouth, United Kingdom) containing ethylenediamine tetraacetic acid. After centrifugation at 2,500 rpm for 15 minutes, plasma samples were collected and stored at-80°C within one day for further enzyme-linked immunosorbent assays (ELISAs) detection. The plasma levels of Ang1, Ang2, and VEGF were determined using ELISAs according to the instructions from the manufacturer (R&D Systems, Minneapolis, Minnesota, United States of America).

### Statistical Analysis

Normality was tested by Shapiro-Wilk test. Continuous variables with normal distribution were expressed as mean±standard deviation and compared by Student’s *t*-test or one-way analysis of variance. Categorical variables were expressed as percentages (number and %) and compared by Chi-squared test. All potential predictors for short-term (within 30 days after surgery) mortality were studied using univariable logistic regression analysis. Correlations were determined using Pearson’s correlation method. For all tests, *P*<0.05 was considered statistically significant.

Statistical analysis was performed using GraphPad Prism Software (Version 8, La Jolla, California, United States of America) and IBM Corp. Released 2013, IBM SPSS Statistics for Windows, version 22.0, Armonk, NY: IBM Corp.

## RESULTS

### Patients’ Characteristics

Table 1 shows the demographic data of the patients enrolled in this study. Patients with VHD consisted of 22 men and 20 women (age: 55.65±1.71 years). We retrieved information, including sex, age, New York Heart Association (NYHA) classifications, ejection fraction (EF), N-terminal pro B-type natriuretic peptide level, creatinine, uric acid, white blood cell count, neutrophils, neutrophil percentage, neutrophil-to-lymphocyte ratio, platelet, cardiopulmonary bypass duration, aortic cross-clamping duration, length of tracheal intubation, length of intensive care unit stay, and length of hospital stay from patients’ hospital charts.

**Table 1:** Clinical characteristics of VHD patients (n=42).

Variables	n (%) or mean±SD
Age (years)	55.65±1.71
Male/female (% male)	22/20 (52.38%)
NYHA	
I	2 (4.76%)
II	28 (66.67%)
III	10 (23.81%)
IV	2 (4.76%)
EF (before surgery)	63.41±1.54
EF (after surgery)	56.98±1.70
NT-pro-BNP (before surgery)	1412.10±402.41
Creatinine	89.37±4.87
Uric acid	446.00±20.87
WBC count (109/L)	7.00±0.29
Neutrophil (109/L)	4.07±0.24
Neutrophilic percentage	0.57±0.01
Neutrophil-to-lymphocyte ratio	2.07±0.14
PLT	224.34±9.05
CPB duration (min)	146.81±6.71
Aortic cross-clamping duration (min)	84.93±4.03
Length of tracheal intubation (hour)	33.13±15.52
Length of ICU stay (day)	2.70±0.65
Length of hospital stay (day)	29.74±1.73

The results are expressed as n (%) or mean±standard deviation (SD) CPB=cardiopulmonary bypass; EF=ejection fraction; ICU=intensive care unit; NT-pro-BNP=N-terminal pro B-type natriuretic peptide; NYHA=New York Heart Association; PLT=platelet; VHD=valvular heart disease; WBC=white blood cell

### Concentration of Ang1, Ang2, and VEGF in VHD Patients with Different NYHA Classifications

Ang1, Ang2, and VEGF levels were detected in the plasma samples of VHD patients and the healthy control group (Tables 2 and 3). Compared with the healthy control group, Ang2 was significantly decreased in the VHD group (control = 1736.70±95.69 pg/mL, VHD = 1365.05±98.48 pg/mL, *P*=0.023), while no significant difference was observed in the Ang1 and VEGF levels. Further, differences in plasma Ang1 and Ang2 and VEGF levels among patients with NYHA I/II classes, NYHA III/IV classes, and controls were analyzed. Interestingly, only the Ang2 expression level of NYHA I/II patients was significantly decreased compared with that of healthy control individuals, but the same was not true for NYHA III/IV patients (NYHA I/II: 1317.01±120.36 pg/mL *vs.* healthy control group: *P*=0.017; NYHA III/IV: 1567.81±189.27 pg/mL *vs.* healthy control group: *P*=0.485), suggesting that decreased Ang2 might be associated with better cardiac function in VHD.

**Table 2:** Ang1, Ang2, and VEGF levels in control group and VHD patients.

Parameter	Control (n=20)	Total VHD (n=42)	*P*-value
Ang1 (pg/mL)	2842.96±769.51	6147.63±1894.10	0.112
Ang2 (pg/mL)	1736.70±95.69	1365.05±98.48	**0.023**
VEGF (pg/mL)	109.58±17.92	110.86±14.63	0.956

The results are expressed as means±standard deviation. Differences in the data between the two groups were determined by Student’s *t*-test Ang=angiopoietin; VEGF=vascular endothelial growth factor; VHD=valvular heart disease

**Table 3:** Ang1, Ang2, and VEGF levels in control group and VHD patients.

Parameter	Control (n=20)	VHD with NYHA I/II (n=30)	VHD with NYHA III/IV (n=12)	*P*-value^a^	*P*-value^b^
Ang1 (pg/mL)	2842.96±769.51	7841.771±2927.14	3602.88±769.35	0.151	0.857
Ang2 (pg/mL)	1736.70±95.69	1317.01±120.36	1567.81±189.27	**0.017**	0.485
VEGF (pg/mL)	109.58±17.92	121.02±20.87	88.34±44.70	0.686	0.531

The results are expressed as means±standard deviation. Statistical difference between the groups was evaluated using one-way analysis of variance *P*-value^a^: NYHA I/II *vs.* control *P*-value^b^: NYHA III/IV *vs.* control Ang=angiopoietin; NYHA=New York Heart Association; VEGF=vascular endothelial growth factor; VHD=valvular heart disease

### Univariable Logistic Regression

Univariable analysis (Table 4) showed significant differences between short-term survivors and non-survivors in the following factor: Ang2 (odds ratio 18.75, *P*=0.033, 95% confidence interval 8.08-102.33).

**Table 4:** Univariable logistic regression.

	Univariable analysis		
	OR	95% CI	*P*-value
EF (before surgery) (%)	1.005	(1.001, 1.014)	0.427
EF (after surgery) (%)	1.007	(1.001, 1.016)	0.238
Angiopoietin 1 (pg/mL)	1.087	(1.008, 1.193)	0.113
Angiopoietin 2 (pg/mL)	18.75	(8.08, 102.33)	0.033
Vascular endothelial growth factor (pg/mL)	1.014	(0.994, 1.031)	0.146
CPB duration (min)	1.001	(0.999, 1.005)	0.135
Aortic cross-clamping duration (min)	1.007	(0.989, 1.022)	0.097

Logistic regressions were used to analyze the predictors of 30-day mortality after surgery CI=confidence interval; CPB=cardiopulmonary bypass; EF=ejection fraction; OR=odds ratio

### Correlation of Ang1, Ang2, and VEGF Levels

Given the complex connection of Ang1, Ang2, and VEGF^[14]^, the correlation of Ang1, Ang2, and VEGF levels was also analyzed and is shown in Table 5. Ang1 levels were negatively correlated with Ang2 levels (*P*=0.032, Pearson’s correlation coefficient =-0.317) and positively correlated with VEGF levels (*P*=0.019, Pearson’s correlation coefficient = 0.359).

**Table 5:** Correlations among plasma levels of Ang1, Ang2, and VEGF in VHD patients.

Variables	Ang1	Ang2	VEGF
Ang1			
Pearson’s correlation coefficient	1.000		
*P*-value	--		
Ang2			
Pearson’s correlation coefficient	**-0.317**	1.000	
*P*-value	**0.034**	--	
VEGF			
Pearson’s correlation coefficient	**0.359**	0.177	1.000
*P*-value	**0.019**	0.285	--

Correlations between Ang1, Ang2, and VEGF expression levels were assessed with the parametric Pearson’s rank correlation test. *P*-value < 0.05 was considered statistically significant Ang=angiopoietin; VEGF=vascular endothelial growth factor; VHD=valvular heart disease

## DISCUSSION

In the present study, we found that the plasma level of Ang2 was significantly lower in all VHD patients and VHD patients with NYHA I/II classes — but not in VHD patients with NYHA III/IV classes — than in healthy controls. It may suggest an association of decreased Ang2 levels and better cardiac function and prognosis in VHD. The Ang1 expression level was negatively associated with Ang2 but positively associated with VEGF.

The Ang-Tie axis, one of the most important switch signaling pathways for angiogenesis, has been documented in a wide range of cardiovascular diseases and inflammatory diseases^[1,15]^. Among the pathway members, the best characterized are Ang1 and Ang2. Ang2 mediates vascular leakage^[4]^ and inflammation^[5]^, while Ang1 has antipermeability^[4]^, anti-inflammatory^[6]^, and cardioprotective effects^[7]^ because of their opposite pathophysiological effects in response to binding to the Tie2 receptor^[16]^. Circulating Ang2 levels and the Ang2/Ang1 ratio could serve as suitable biomarkers of inflammatory disease. Circulating Ang2 levels were correlated with markers of endothelial cell activation and 28-day mortality in patients with severe sepsis^[15]^. In acute lung injury patients, the Ang2/Ang1 ratio was an independent predictor of mortality^[17]^. Higher Ang2 and Ang2/Ang1 were associated with poor cardiac function in acute myocardial infarction patients^[18]^. Our results are consistent with previous results. In the present study, lower Ang2 levels were observed in the VHD patients with lower NYHA classes and higher EFs (Table 3). As Ang2 is an important inflammation-associated cytokine^[19,20]^, it may suggest that lower Ang2 levels might prevent VHD progression and improve prognosis by regulating inflammation levels. The association of Ang2/Ang1 ratio and VHD patient characteristics was not observed in the present study (data not shown); however, a significantly negative correlation of Ang1 and Ang2 was observed (Table 5), which might have been due to the small sample size of our study. Furthermore, Ang1 and Ang2 have been shown to interact with VEGF to induce an inflammatory response^[21]^. Our data showed the positive association of Ang1 and VEGF (Table 5), indicating that a synergistic effect of Ang1 and VEGF might exist in VHD, which may regulate the inflammation level. Taken together, these results suggest that Ang2 could affect the disease progression and prognosis of VHD by regulating the inflammatory level via the interaction of Ang1 and VEGF.

In summary, a lower Ang2 level was associated with a better NYHA class in VHD. Univariable logistic regression analysis result indicated that Ang2 was a significant independent predictor for short-term mortality. Furthermore, the association of Ang1, Ang2, and VEGF was found in VHD. Thus, Ang2 might affect the disease progression and prognosis of VHD, which may involve the interaction of Ang1 and VEGF.

### Limitations

As a single-center retrospective study, this study has all the limitations of a retrospective observational study. Thus, as an observational study, cause-effect relationships could not be established. Further prospective studies are necessary to confirm our present findings. Also, the sample size of our study was small. The changes in plasma levels of Ang1, Ang2, and VEGF after valve replacement surgery were not evaluated. Future studies could examine whether the expression of these proteins changes after surgery and how the levels correlate with the outcomes of valve replacement.

## CONCLUSION

Ang2 might serve as a therapeutic and prognostic target for VHD.
